# Lubricating properties of chewing stimulated whole saliva from patients suffering from xerostomia

**DOI:** 10.1007/s00784-020-03758-8

**Published:** 2021-03-04

**Authors:** Jeroen Vinke, Marijn Oude Elberink, Monique A. Stokman, Frans G. M. Kroese, Kamran Nazmi, Floris J. Bikker, Henny C. van der Mei, Arjan Vissink, Prashant K. Sharma

**Affiliations:** 1 Department of Biomedical Engineering, University of Groningen and University Medical Center Groningen, Antonius Deusinglaan 1, 9713AV, Groningen, The Netherlands; 2 Department of Radiation Oncology, University of Groningen and University Medical Center Groningen, Groningen, The Netherlands; 3 Department of Rheumatology and Clinical Immunology, University of Groningen and University Medical Center Groningen, Groningen, The Netherlands; 4 Department of Oral Biochemistry, Academic Centre for Dentistry Amsterdam, Free University and University of Amsterdam, Amsterdam, The Netherlands; 5 Department of Oral and Maxillofacial Surgery, University of Groningen and University Medical Center Groningen, Groningen, The Netherlands

**Keywords:** Sjögren’s syndrome, Radiotherapy, Oral tribology, Salivary lubrication

## Abstract

**Objectives:**

The study aimed to quantify the lubricating properties of chewing stimulated whole saliva from healthy controls (*n* = 22), from patients suffering from primary Sjögren’s syndrome (*n* = 37) and from patients undergoing head-and-neck radiotherapy (*n* = 34).

**Materials and methods:**

All participants had to complete the Xerostomia Inventory questionnaire to score dry mouth sensation. Lubrication was measured using an ex vivo tongue-enamel friction system in terms of Relief and Relief period. MUC5b and total protein concentrations of the saliva samples were measured by an enzyme-linked immunosorbent assay and a bicinchoninic acid assay, respectively.

**Results:**

Relief of Sjögren’s patients’ saliva and post-irradiation patients’ saliva was similar compared with healthy controls, but saliva from post-irradiation patients lubricated significantly better than saliva from Sjögren’s patients. The Relief period was similar between the three groups. The Relief and Relief period were higher for saliva samples post-irradiation compared to pre-irradiation. MUC5b and total protein concentrations were comparable in all groups. MUC5b and total protein output were significantly lower in patients subjected to radiotherapy compared to saliva from healthy controls and pre-irradiation patients. MUC5b concentrations positively correlated with lubricating properties of post-irradiation patient saliva.

**Conclusions:**

The lubricating properties of patient saliva were not any worse than healthy controls. Lower flow rate leads to lower availability of saliva in the oral cavity and decreases the overall output of protein and MUC5b, which might result in an insufficient replenishing of the mucosal salivary film.

**Clinical relevance:**

An insufficient replenishing might underlie the sensation of a dry mouth and loss of oral function.

**Supplementary Information:**

The online version contains supplementary material available at 10.1007/s00784-020-03758-8.

## Introduction

Xerostomia is a common complaint of, amongst others, patients suffering from Sjögren’s syndrome, patients with diabetes type II, patients using multiple drugs and patients treated with radiotherapy in the head and neck region [1–4]. Besides having difficulty in speech, mastication and swallowing, patients with a reduced salivary secretion have an increased risk of developing rapid progressing dental caries and oral infections. All these factors contribute to poor quality of life.

Xerostomia is most commonly associated with a reduced salivary flow rate, although the flow rate can be normal in sufferers [5]. Also associated with xerostomia is a decreased water-retaining ability of salivary mucins [6–8]. There are two types of salivary mucins, namely MUC5b and MUC7. MUC5b is a long-chain, heavily glycosylated protein that is able to form large gel-like networks due to polymerization via the cysteine-rich domains in the peptide backbone [9]. The main aspects that determines the lubricating properties of salivary mucins are the glycosylation density and specific water retaining end groups like sialic acid residues (by electrostatic interactions) and sulphated end groups (by H-bonds and electrostatic interactions) [10, 11]. So, mucins provide lubrication within the oral cavity and its properties determine the composition and thickness of the residual salivary film [12]. Decreased water-retention properties have negative consequences for the lubricating properties of saliva [13–15]. Furthermore, an insufficient salivary film thickness is linked to dry mouth sensation [16].

Differences in lubricating properties of saliva might be an explanatory factor for difficulty in speech, mastication and swallowing and actual dry mouth sensation in patients with xerostomia. Therefore, this study aimed to compare lubricating properties of saliva from healthy controls and two well-defined groups of xerostomia patients, i.e. patients suffering from Sjögren’s syndrome and patients that had been subjected to head and neck radiotherapy. The lubricating properties of saliva were measured using the ex vivo tongue-enamel friction system developed by Vinke et al. [17], in terms of ‘Relief’ and ‘Relief period’.

## Materials and methods

### Cohorts

A cohort of healthy controls (HC, *n* = 22), a cohort of patients with dry mouth complaints due to primary Sjögren’s syndrome (pSS, *n* = 37) and a cohort of patients that were subjected to head and neck radiotherapy (*n* = 34) were included in this study.

In the healthy control group, volunteers from roughly the same age category as the two patient’s groups were included. Volunteers were recruited from the Department of Biomedical Engineering at the University Medical Center Groningen. A routine clinical examination revealed that these healthy controls did not smoke, did not use medication (anticonception was allowed) and did not have a history with autoimmune diseases, head and neck radiotherapy or chemotherapy.

Patients with dry mouth complaints who visited the Department of Rheumatology and Clinical Immunology for a diagnostic Sjögren’s syndrome work-up were asked to join the study. Included were patients that on completion of the diagnostic work-up fulfilled the classification criteria of the American College of Rheumatology and the European League Against Rheumatism for Sjögren’s syndrome [18]. Patients with negligible stimulated whole saliva secretion (< 0.01 ml/min) were excluded because minimally required volumes for experiments cannot be reached.

Patients being treated with primary radiotherapy for tumours in the head and neck region ((supra)glottis laryngeal, oropharyngeal, hypopharyngeal, tonsil, base of tongue) were recruited from the Department of Radiation Oncology of the University Medical Center Groningen. Patient treatment was curative and performed by primary standard-fractionated intensity-modulated radiotherapy (dose of 2 Gy at five consecutive days of the week for 7 weeks, 70 Gy in total). As part of the routine diagnostic work-up treatment evaluation, stimulated whole saliva was collected before and 6 months after radiotherapy.

For radiotherapy patients, medication usage was noted on both visits, either before or after radiotherapy treatment. pSS patients were seen only once, and so, medication usage was only noted once. Medication was screened for causing xerogenic side effects [4]. Medication usage was divided into two categories: (1) zero to four different drugs without known xerogenic side effects and (2) over four drugs (polypharmacy) or at least one xerogenic drug.

Approval for this study was obtained from the Medical Ethics Review Board of the University Medical Center Groningen (M17.2157256, M12.114967). All participants gave informed consent. STROBE guidelines were followed during this study.

### Whole saliva collection and treatment

Saliva was collected from all subjects by an oral hygienist trained in collecting whole saliva between 9 and 12 a.m. to minimize circadian effects on saliva flow. Participants were asked to refrain from eating and drinking 1 h prior to collection, except from drinking water. Before collection, the mouth was rinsed with tap water. Participants were asked to sit straight and not to speak and swallow during collection. Saliva stimulation was achieved by chewing on Parafilm® (2.5 × 5 cm) for 5 min. Saliva was accumulated in the floor of the mouth, and the subject spat it into the pre-weighed container every 60 s or as soon as the patient experiences an urge to swallow the fluid accumulated in the floor of the mouth. Samples were collected on ice. Saliva-filled containers were weighed to calculate the salivary flow rate by assuming saliva density of 1 g/ml. The saliva of healthy controls and radiotherapy patients was centrifuged for 5 min at 10,000*g* (Beckman Coulter Avanti® J-E centrifuge, Fullerton, CA, USA) at 10 °C. An amylase inhibitor (phenylmethylsulfonylfluoride, Sigma-Aldrich, St Louis, MO, USA) was added to the supernatant to a final concentration of 1 mM, and aliquots were frozen in liquid nitrogen and stored at − 80 °C until analysis.

### Xerostomia Inventory questionnaire

The validated Dutch-version of the Xerostomia Inventory [19, 20] was completed by the participants. The Xerostomia Inventory contains eleven questions related to subjective oral dryness using an ordinal scoring scale ranging from 11 to 55 (Fig. 5 in the Supplementary material).

### Salivary lubrication

Salivary lubrication was tested using the ex vivo tongue-enamel friction system [17]. In short, the friction of bovine tooth enamel on a porcine tongue was measured during reciprocating sliding using a universal mechanical tester (UMT-3, CETR Inc., Billerica, MA, USA) (Fig. 6 in the Supplementary material). For the first 10 cycles, friction force (F_friction_) was measured in dry condition, to mimic xerostomic conditions. Then, 20 μl of saliva was brought in the tooth-enamel sliding interface and continued to monitor the frictional forces. The normal force (F_normal_) at the tongue-enamel interface was kept constant at 0.25 N. The ratio of the friction force to normal force was calculated to get the coefficient of friction (μ = F_friction_/F_normal_). The ratio of μ_dry_/μ_lubricated_ was reported as ‘Relief’. The Relief was calculated based on the maximum (Relief_max_) and median (Relief_med_) friction coefficient from each friction cycle. The duration for which the μ_lubricated_ remained low was taken as ‘Relief period’. The pre-set sliding velocity was 4 mm/s; the sliding distance was 10 mm. Relief_max_, Relief_med_ and Relief period were collectively termed as saliva lubricating parameters.

### Salivary lubricating of different volumes of saliva of radiotherapy patients

From two of the radiotherapy patients, different volumes of saliva were tested. Volumes of saliva tested were 5, 10, 20, and 25 μl. These volumes were applied on the same predefined surface area (233 mm^2^; Supplementary Figure 6). Saliva samples were collected before and 6 months after radiotherapy in two male patients (45 and 69 years of age). Patients had not been subjected to a surgical dissection of the tumour. The patients did not use xerogenic medication, either before or after radiotherapy.

### Protein analysis

Total protein (further referred to as protein) concentration in saliva was analysed using bicinchoninic acid (BCA) assay (Pierce BCA protein assay kit, Thermo Scientific, Rockford, IL, USA) following the manufacturer’s protocol using bovine serum albumin as a standard as described earlier [21].

Enzyme-linked immunosorbent assay (ELISA) was performed to measure the MUC5b as described before [22]. Saliva was diluted in coating buffer (100 mM Na_2_CO_3_, pH 9.6) to a final concentration of 5 μl/ml and then twofold serially diluted and incubated in microtiter plates in duplicate overnight at 4 °C. Plates were washed with 2% Tween-20 in phosphate-buffered saline (PBS-T). The microtiter plates were blocked with a 1% gelatine solution in PBS-T (PBS-T-gelatine), during 1 h under shaking at 37 °C. 25 μl/ml of monoclonal F2 antibody (ACTA, Amsterdam, Netherlands) in PBS-T-gelatine was added and incubated for 1 h at 37 °C and washed with PBS-T. F2 recognizes the sulfo-Lewis antigens on salivary MUC5b. 0.5 μl/ml polyclonal rabbit anti-mouse immunoglobulin HP-conjugate (Dakopatts, Glostrup, Denmark) in PBS-T-gelatine was added and incubated for 1 h at 37 °C. After washing with PBS-T, a colouration reaction was initiated by adding 25 μl/ml TMB (3,3, 5,5,-tetramethylbenzidine, Merck, Darmstadt, Germany) in PBS-T-gelatine with 1 μl/ml H_2_O_2_ for 15 min at room temperature. The reaction was stopped with 50 μl H_2_SO_4_ (2 M) per well. The absorbance was measured at 450 nm with an ELISA plate reader. MUC5b concentration was reported in arbitrary units (a.u.) per ml. Every plate contained the same control, pooled healthy whole stimulated saliva diluted 200 times in coating buffer. MUC5b output and protein output were reported in a.u./min and mg/min, respectively.

### Statistical analysis

A power analysis was performed using an estimated sigma and delta of 4, alpha of 0.05 and a beta of 0.1. The power was 0.9. The estimated group size was 22 individuals per group. An additional number of 12 patients were added to the patient groups to compensate for the expected dropout rate. Group statistics were performed using Graphpad Prism5.0 software. Non-normally distributed data were analysed using Kruskal-Wallis test with Dunn’s multiple comparison test. Normally distributed data were analysed using 1-way analysis of variance with Bonferroni’s multiple comparison test. Paired data, prior to and after radiotherapy, were analysed with Wilcoxon-signed rank tests or paired Student’s *t* tests depending on normality. Normality was tested using Q-Q plots. Pearson’s correlation analyses were performed to correlate parameters. Multivariate regression analysis (SPSS 25) was performed to analyse significant influences of age, gender, medication intake, disease (pSS or radiotherapy) and MUC5b and protein concentration on the lubrication parameters and dry mouth score. The multivariate regression analysis makes linear regression analyses for each condition, after the influence of each condition is normalized. Influences of cofactors in the analysis are eradicated and deliver the importance of each condition regarding lubrication parameters and dry mouth.

## Results

### Patients and healthy controls

Distribution of age and gender, stimulated flow rate, Xerostomia Inventory, and the category of medication usage are given in Table 1. Xerogenic medication and the occurrence are shown in Table 3 in the supplementary data. The radiotherapy group initially had 34 patients prior to radiotherapy of which 24 returned for saliva sampling 6 months after radiotherapy. Patient dropout was due to a too limited salivary secretion (*n* = 2), tumour recurrence (*n* = 2), being deceased (*n* = 4) or other reasons (*n* = 2). Data of the 24 participants that returned after completion of radiotherapy (after radiotherapy group; 70.5% response) were compared with their before radiotherapy levels. The mean baseline salivary flow rates and the Xerostomia Inventory scores were comparable between the 24 radiotherapy patients who returned after radiotherapy and the 10 patients that were lost to follow-up.

**Table 1:** Characteristics of the study groups healthy controls (HC), primary Sjögren’s syndrome (pSS) and before (BRT) and after radiotherapy (ART)

Cohort	Number	Age	Male-female ratio	Flow rate^#^ (ml/min)	Xerostomia Inventory^$^	Cat. xerogenic medication
HC	22	50.1 ± 14.0	9:13	1.41 ± 0.79	12 (11–14)	22:0
pSS	37	52.4 ± 13.6	6:31	0.82 ± 0.54	27 (21–38)	13:24
BRT	24*	63.3 ± 8.6	19:5	1.06 ± 0.54*	11 (11–12)*	8:16
ART	24	63.9 ± 8.6	19:5	0.50 ± 0.31	16 (13–22)	6:15 (3 dd)^#^

^#^Flow rate is the chewing stimulated whole salivary flow rate and is also displayed in Figs. 1a and 2a. ^$^Xerostomia Inventory scores displayed as median values with the interquartile ranges in between brackets and are also displayed in Figs. 1b and 2b. *10 patients prior to radiotherapy were lost to follow-up. The mean saliva flow rates and the Xerostomia Inventory scores were comparable between the 10 patients prior to radiotherapy lost to follow-up and the 24 patients prior to radiotherapy in whom 6 months after radiotherapy a second saliva samples was obtained (after radiotherapy). #For three persons there was a data deficiency (dd).

**Fig. 1:**
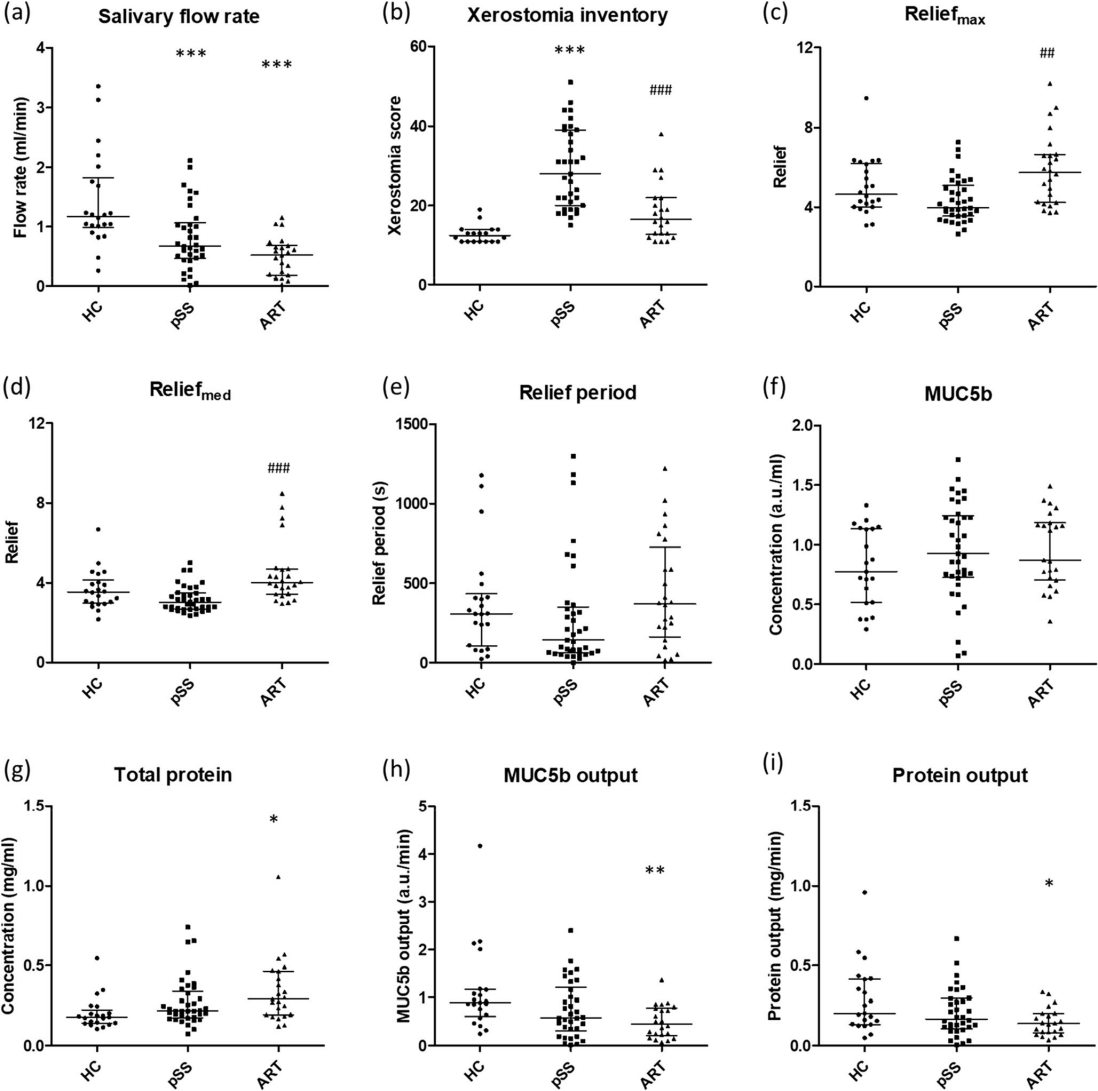
Differences in saliva characteristics and dry mouth sensation of healthy controls (HC), primary Sjögren’s syndrome patients (pSS) and head and neck radiotherapy patients after radiotherapy (ART). **a** Flow rate of stimulated whole saliva. **b** Xerostomia Inventory scores. Measured Relief_max_ (**c**) and Relief_med_ (**d**) when adding 20 μl of saliva to the tongue-enamel friction system. **e** Relief period. **f** MUC5b concentration. **g** Protein concentration. **h** MUC5b output. **i** Protein output. Statistical differences compared to HC are marked by * (p < 0.05), ** (p < 0.01) or *** (p < 0.001). The symbol ^#^ indicates significant differences between ART and pSS. Error bars represent the interquartile ranges and median value

Whole stimulated salivary flow rate was significantly lower in patients compared to healthy controls (Fig. 1a). The whole stimulated salivary flow rate of the patients prior to radiotherapy was lower compared with healthy volunteers. The Xerostomia Inventory score of pSS was significantly higher compared to healthy controls and patients treated with radiotherapy (Fig. 1b). The lubricating properties as measured by the tongue-enamel friction system, Relief_max_ and Relief_med_, of saliva from patients with pSS and patients being treated with radiotherapy patients were found to be comparable with healthy controls (Fig. 1c, d). The Relief_max_ and Relief_med_ provided by saliva of patients treated with radiotherapy were significantly higher than in saliva of pSS patients. No statistical significant differences were found in Relief period and MUC5b concentration between the three groups (healthy controls, pSS, after radiotherapy) (Fig. 1e, f). Protein concentration in saliva from patients treated with radiotherapy was found to be significantly higher than the protein concentration in saliva from healthy controls (Fig. 1g). The MUC5b output and protein output (concentration × salivary flow rate) were significantly lower in patients treated with radiotherapy compared with healthy controls (Fig. 1h, i).

### Comparison of before and after radiotherapy

**Fig. 2:**
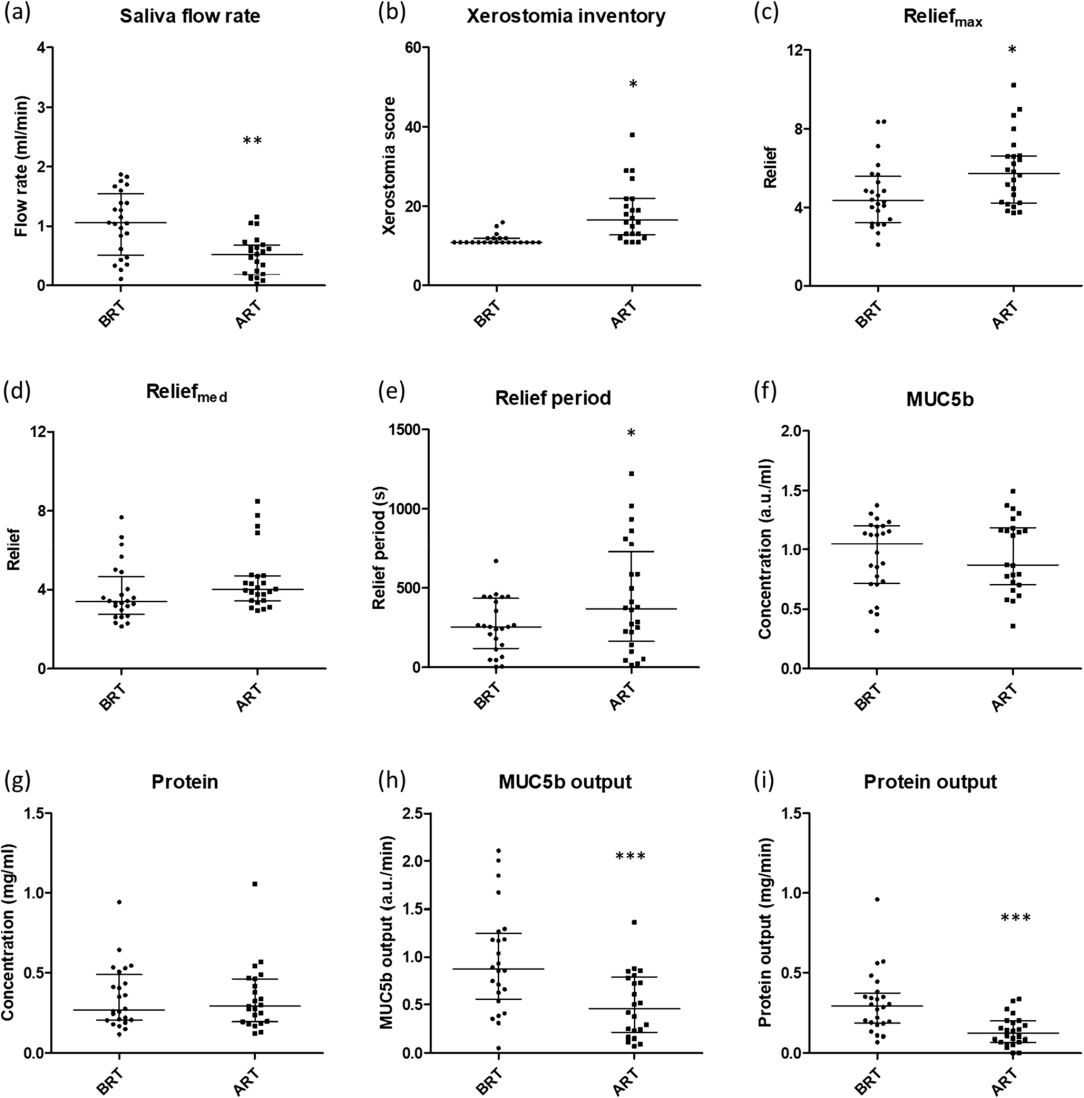
Differences in saliva characteristics and dry mouth sensation of patients before (BRT) and after (ART) radiotherapy treatment. **a** Stimulated whole salivary flow rate. **b** Xerostomia Inventory scores. Measured Relief_max_ (**c**) and Relief_med_ (**d**) by adding 20 μl of saliva to the tongue-enamel friction system. **e** Relief period. **f** MUC5b concentration. **g** Protein concentration. **h** MUC5b output. **i** Protein output. Statistical differences are shown by * (p < 0.05), ** (p < 0.01 or *** (p < 0.001). Paired statistics were performed. Error bars represent the interquartile ranges and the median value

Stimulated whole salivary flow rate decreased significantly post-irradiation (Fig. 2a) while the Xerostomia Inventory score increased significantly post-irradiation (Fig. 2b). Relief for patients prior to radiotherapy was comparable with healthy controls. Relief_max_ and the Relief period were significantly higher after radiotherapy compared with prior to radiotherapy (Fig. 2c, e). No significant differences were found in Relief_med_ (Fig. 2d). MUC5b and protein concentrations did not differ between patients prior to an after radiotherapy (Figs. 2f, g) in contrast to MUC5b and protein output which were significantly lower for patients after radiotherapy (Fig. 2h, i). Medication intake for patients treated with radiotherapy slightly differed between both visits (Supplementary table 3).

### Correlations

**Fig. 3:**
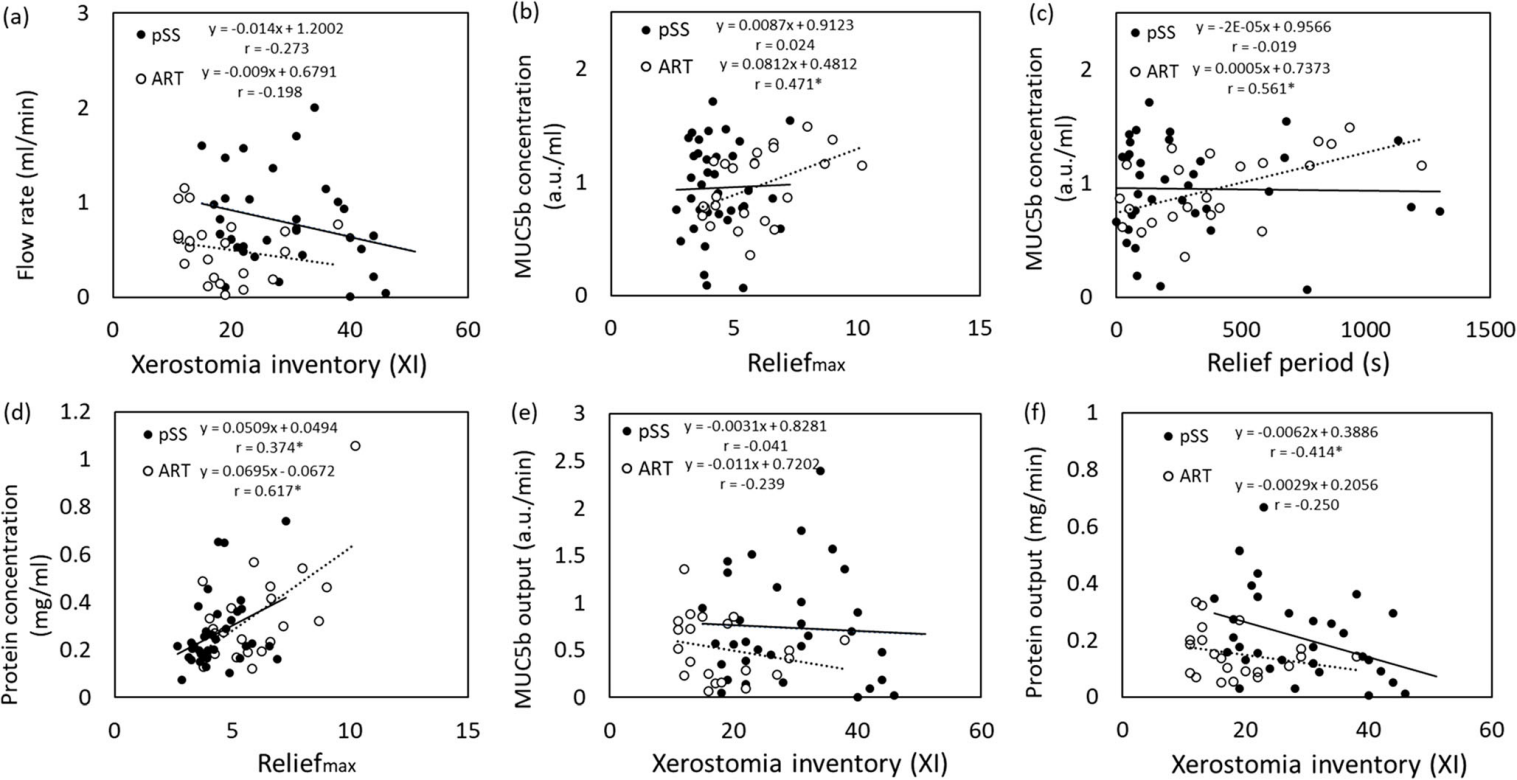
Correlation of saliva characteristics and dry mouth sensation in primary Sjögren’s syndrome patients (pSS) and patients that were treated with radiotherapy (ART). **a** Flow rate as a function of the Xerostomia Inventory score. **b** MUC5b concentration as a function of Relief _max_ and **c** MUC5b concentration as a function of Relief period. **d** Protein concentration as a function of Relief_max_. **e** MUC5b output in saliva from patients that were treated with radiotherapy as a function of the Xerostomia Inventory score. **f** Protein output as a function of the Xerostomia Inventory score. Pearson’s correlation coefficient r is displayed in each graph. Asterisk = correlation was significant (p < 0.05)

Whole salivary flow rate did not correlate significantly with the Xerostomia Inventory score in patients treated with radiotherapy (*p* = 0.378) and patients with pSS (*p* = 0.145) (Fig. 3a). A significant correlation was found between MUC5b concentration and Relief_max_ (*p* = 0.023) and Relief period (*p* = 0.005) in saliva from patients treated with radiotherapy (Fig. 3b, c), while protein concentration correlated with Relief_max_ in both patients with pSS and patients treated with radiotherapy (*p* = 0.023 and *p* = 0.002 respectively) (Fig. 3d). MUC5b output did not correlate significantly with the Xerostomia Inventory score (*p* = 0.828 and *p* = 0.296 for patients with pSS and patients treated with radiotherapy respectively) (Fig. 3e), in contrast to protein output which correlated with the Xerostomia Inventory score in pSS (*p* = 0.023) (Fig. 3f).

### Volume dependency on Relief before and after radiotherapy

**Fig. 4:**
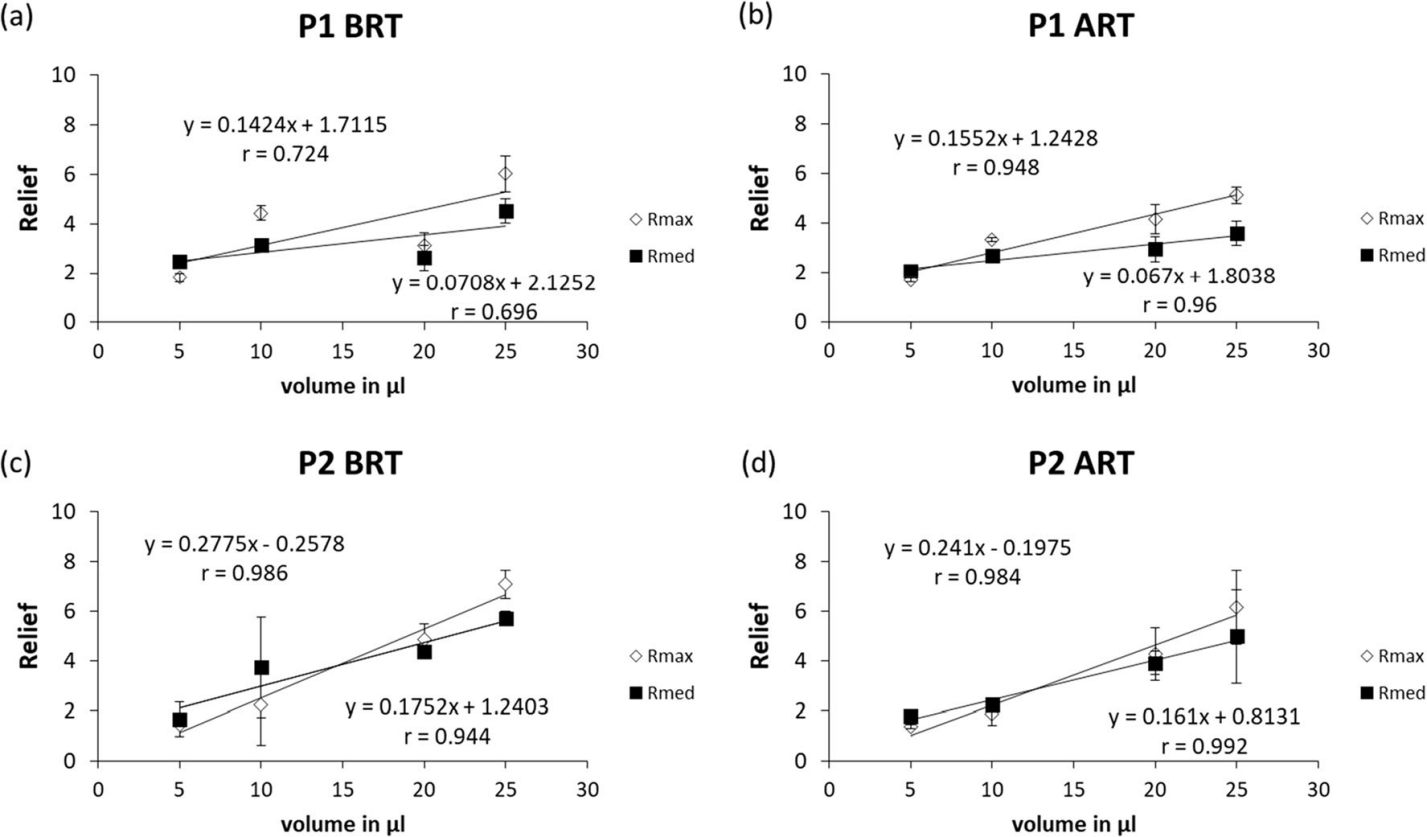
Relief_*med*_ and Relief_*max*_ of various volumes of saliva from two patients (P1 and P2) collected before radiotherapy (BRT) and 6 months after radiotherapy (ART). P1 **a** before and ***b*** after radiotherapy, and *P2* **c** before and ***d*** after radiotherapy. The equations for the slopes of Relief as a function of volume are displayed in the graphs. Error bars represent the standard deviation of duplicate measurements

The Xerostomia Inventory score of both patients was 11 before radiotherapy. After radiotherapy treatment, these Xerostomia Inventory scores of patients 1 and 2 were 12 and 18, respectively. The cumulative radiation doses on the left and right submandibular glands of patient 1 were 48.4 Gy and 46.8 Gy, respectively, and the cumulative doses on the left and right parotid glands 18.2 Gy and 18.8 Gy, respectively. Patient 2 received cumulative radiation on the left and right submandibular gland of 55.2 Gy and 55.0 Gy, respectively, and a cumulative dose on the left and right parotid glands of 28.8 Gy and 32.4 Gy, respectively. The flow rate of stimulated whole saliva of patient 1 dropped from 1.76 ml/min before radiotherapy to 1.15 ml/min after radiotherapy. The salivary flow rate of patient 2 dropped from 1.15 ml/min before radiotherapy to 0.14 ml/min after radiotherapy. The mucin concentration for patient 1 did not differ between before and after radiotherapy (1.14 a.u./ml, 1.18 a.u./ml respectively), while protein concentration had decreased (0.55 mg/ml before radiotherapy, 0.29 mg/ml after radiotherapy). For patient 2, both MUC5b (0.78 a.u. before radiotherapy, 1.12 a.u. after radiotherapy) and protein concentrations (0.11 mg/ml before radiotherapy, 0.38 mg/ml after radiotherapy) increased. For patient 1, the MUC5b output decreased with 32% (from 2.00 a.u./min before radiotherapy to 1.36 a.u./min after radiotherapy) and protein output decreased with 66% (from 0.96 mg/min before radiotherapy to 0.33 mg/min after radiotherapy). For patient 2, the MUC5b secretion rate decreased with 82% (from 0.89 before radiotherapy to 0.16 after radiotherapy) and protein secretion rate decreased with 59% (from 0.13 before radiotherapy to 0.054 after radiotherapy). Relief_med_ and Relief_max_ of different volumes of saliva from both patients are shown in Fig. 4. The slope of Relief per volume is highly comparable between before radiotherapy and after radiotherapy within the same patient.

### Multivariate regression analysis

Multivariate regression analysis (Table 2) showed that protein concentration had a major influence on Relief (*p* < 0.001 for Relief_max_ and Relief_med_). Furthermore, protein output (*p* = 0.027) had a strong influence on the Xerostomia Inventory score. Having been treated with radiotherapy or suffering from pSS influences the Xerostomia Inventory score. According to the multivariate regression analysis, age, gender and salivary flow rate influenced neither the saliva lubricating parameters nor the Xerostomia Inventory score.

**Table 2:** Significant influence of the different parameters on the lubricating properties of saliva and dry mouth sensation (multivariate regression analysis). Parameters were considered significant for *p* < 0.05

Parameter	Relief_max_	Relief_med_	Relief period (min)	Xerostomia Inventory
Age	0.243	0.264	0.761	0.362
Gender	0.661	0.662	0.868	0.940
Salivary flow rate (ml/min)	0.670	0.637	0.907	0.181
MUC5b concentration (a.u./ml)	0.871	0.871	0.916	0.155
MUC5b output (a.u./min)	0.838	0.989	0.954	0.060
Protein concentration (mg/ml)	< 0.001	< 0.001	0.270	0.310
Protein output (mg/min)	0.123	0.117	0.635	0.027
Subject group (healthy, pSS, radiotherapy)	0.307	0.088	0.476	< 0.001
Influence of medication	0.720	0.186	0.983	0.963

## Discussion

In this study, we assessed the lubricating properties of saliva of healthy controls and two well-defined groups of xerostomia patients, pSS and patients treated with radiotherapy. Since we expected that it would be hard to get a sufficient volume of unstimulated whole saliva from all patients within a reasonable time frame, we used stimulated whole saliva in our experiments. In essence, we showed that the lubricating ability of patient saliva was not lower than saliva of healthy controls when the same volume of stimulated saliva (20 μl) was used at the tongue-enamel interface (Fig. 1c, d, e). Furthermore, we proposed that a dry mouth sensation in xerostomia patients might be linked to the lower availability of saliva in the oral cavity and lower MUC5b output.

Preferably, age- and gender-matched groups would have been used; however, since the two patient groups are characterized by different gender ratios and also a different range of ages, it was difficult to match these parameters between groups. Radiotherapy in the head and neck region occurs two times more often in males than in females [23], whereas pSS predominantly affects middle-aged females [24]. Both parameters (age and gender) were taken into account for the multivariate regression analysis and turned out to have insignificant influences on neither the dry mouth parameters, nor the Xerostomia Inventory score.

Lubrication in the oral cavity is provided by saliva. MUC5b is the main glycoprotein in saliva to facilitate lubrication by its water-retaining properties [25], and differences in concentration of MUC5b might influence lubrication parameters [17]. Because of the low salivary flow rates and high Xerostomia Inventory scores of patients with pSS and patients treated with radiotherapy, compared with those of healthy controls and patients prior to radiotherapy, we expected to measure a lower lubrication ability of xerostomia patient saliva in terms of Relief and Relief period. Relief and Relief period of saliva from patients with pSS and patients treated with radiotherapy were, however, not lower than those of saliva from healthy controls. The observation that the Relief and Relief period of healthy controls and the patient groups did not differ could be a result of the comparable MUC5b concentrations in saliva within these groups. The observation that the patients prior to radiotherapy had a lower flow rate compared with healthy controls could be due to the fact that these patients used xerogenic medication already prior to radiotherapy [4].

Our results show that protein concentration correlated positively with Relief (Fig. 3d) which was not in line with what we expected since salivary mucins have been identified to provide the major lubricating properties of saliva. Of course, the total proteins that were measured include the glycoproteins MUC5b and proline-rich proteins. The latter were shown to lubricate better in increasing concentrations [26] and were also shown to be reduced in dry mouth patients [27].

The lubricating properties of saliva from patients with pSS and from patients after radiotherapy as such were not different from that of healthy controls, even though the Xerostomia Inventory score was higher for the patient groups. Patients with pSS and patients treated with radiotherapy had lower salivary flow rates, as well as protein and MUC5b output. Group-wise comparison suggests that a decrease in stimulated whole salivary flow and experiencing xerostomia are interrelated (Figs. 1a, b and 2a, b). The multivariate regression analysis (Table 2) and the insignificant correlation between chewing-stimulated salivary flow rate and the Xerostomia Inventory score in dry mouth patients (Fig. 3a) show that other factors, e.g. MUC5b output, will play a more important role in explaining the dry mouth sensation.

A decreased MUC5B output may lead to insufficient availability of MUC5b for subsequent maintenance and hydration of the salivary film on the oral mucosa [28], as MUC5b is the protein providing lubrication by binding water molecules on its sialylated and sulphated glycan end groups via electrostatic interactions and hydrogen bonds [6, 15]. The salivary film thickness on the oral mucosa showed to be associated with dry mouth sensation [12, 29–31]. Salivary film thicknesses of 10 μm and 30 μm on the palatal mucosa and tongue, respectively, were reported to be threshold values for developing xerostomia [29]. This theory could be valid in radiotherapy-treated patients, given the decreased MUC5b output in these patients (Fig. 1h), as also found by others [28, 32]. It is proposed that in patients treated with radiotherapy, destruction of the superficial epithelial cells of the oral mucosa by irradiation leads to diminished attachment sites for salivary mucins [33]. The lack of mucosal coverage can locally increase friction leading to dry mouth sensation [30]. This could declare the correlation between MUC5b concentrations and Relief and Relief period in the patients treated with radiotherapy that were found in this ex vivo study (Fig. 3b and c). This implies that radiotherapy patients still have functional mucin but not enough for a thick enough layer on the mucosa.

A decreased MUC5b output was not seen for patients with pSS. In patients with pSS, the development of xerostomia has been linked to hypo-sulphation and hypo-glycosylation of MUC5b reducing the water-retaining capacity of MUC5b [6–8, 11, 34]. This might cause an insufficient salivary film thickness [8, 16] and therefore lubricating properties [35]. An increased MUC5b output in patients with pSS would therefore not improve lubrication (Fig. 3b and c).

Overall, no differences in lubricating properties were found between healthy controls and patients with pSS and patients who were treated with radiotherapy when 20 μl of saliva was used in the experimental model. Possible explanations for this observation could be a sufficient amount of MUC5b, irrespective of their water retaining capacities, or by a sufficiently thick layer of saliva that was present in the tongue-enamel interface ex vivo. To test this hypothesis, the lubricating properties of saliva from two radiotherapy patients (both before and after radiotherapy) that had similar protein and MUC5b concentrations, were measured in the tongue-enamel friction system. Different volumes of saliva were applied to vary the salivary film thickness. Application of 5 μl of volume corresponded to an average film thickness of 20 μm on the tongue, while 25 μl corresponds to layer thickness of 100 μm. Figure 4 shows a strong linear relation between Relief and the applied volume of saliva and thus the salivary film thickness. The limited results of this pilot experiment support the thoughts of Wolff and Kleinberg [29] that a low saliva availability will result in a decreased salivary film thickness as well as that a lower MUC5b output possibly decreases the thickness and water retaining ability of the salivary film. These phenomena may result in a dry mouth sensation. In both patients, the slope (rate of decrease in Relief as a function of decrease in saliva volume) remained the same before and 6 months after radiotherapy (Fig. 4). These findings imply that the slope is more dependent on the patient than on the time of sampling or the actual treatment. We could speculate that patients with a higher slope are more sensitive to a decrease in salivary flow rate and would earlier complain of dry mouth, although this hypothesis needs further investigation.

## Conclusions

Salivary lubricating properties of primary Sjögren’s syndrome and radiotherapy patients were similar to saliva from healthy controls. It appears that the salivary lubrication is dependent on mucosal salivary film which depends on sufficient availability of saliva and output of proteins and glycoproteins. Availability of saliva and output of proteins and glycoproteins depends on salivary flow rate amongst other reasons. The significantly reduced MUC5b and protein output in patients after radiotherapy possibly led to a reduced salivary film thickness. Therefore, dry mouth sensation of xerostomia patients might be explained by lower availability of saliva in the oral cavity leading to lower MUC5b and proteins in the mucosal salivary film.

## Supplementary Information

ESM 1 (DOCX 1.92 mb)
