# Supplementary material for: Lubricating properties of chewing stimulated whole saliva from patients suffering from xerostomia
Source: Clin Oral Investig. 2021 Mar 4;25(7):4459–69. doi: 10.1007/s00784-020-03758-8 (PMC8310523; doi:10.1007/s00784-020-03758-8)
Supplement: Supplementary file 1 — (DOCX 1.92 mb) [file 784_2020_3758_MOESM1_ESM.docx]

Supplementary Material

## Lubrication properties of chewing stimulated whole saliva from patients with xerostomia

Jeroen Vinke^1^, Marijn Oude Elberink^1^, Monique A. Stokman^2^, Frans G.M. Kroese^3^, Kamran Nazmi^4^, Floris J. Bikker^4^, Henny C. van der Mei^1^, Arjan Vissink^5^, Prashant K. Sharma^1^*

^1^ Department of Biomedical Engineering, University of Groningen and University Medical Center Groningen, Antonius Deusinglaan 1, 9713AV, Groningen, The Netherlands

^2^ Department of Radiation Oncology, University of Groningen and University Medical Center Groningen, Groningen, The Netherlands

^3^ Department of Rheumatology and Clinical Immunology, University of Groningen and University Medical Center Groningen, Groningen, The Netherlands

^4^ Department of Oral Biochemistry, Academic Centre for Dentistry Amsterdam, Free University and University of Amsterdam, Amsterdam, The Netherlands

^5^ Department of Oral and Maxillofacial Surgery, University of Groningen and University Medical Center Groningen, Groningen, The Netherlands


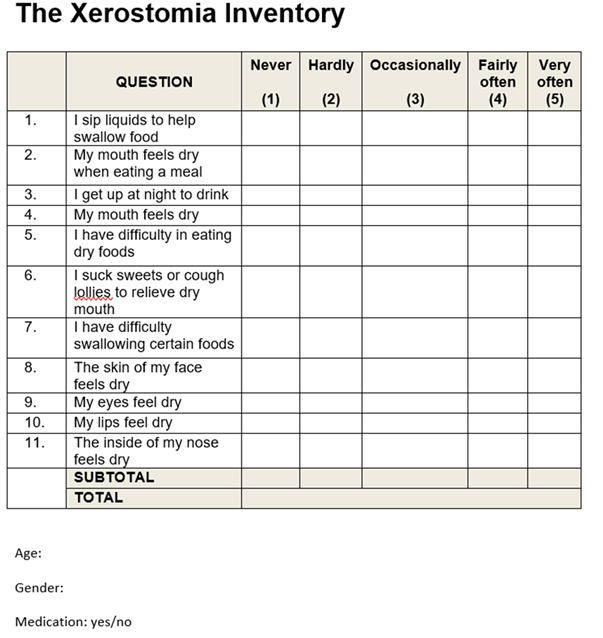


Supplementary Fig. 5 The Xerostomia Inventory. A validated Dutch version was used in the study [1, 2]


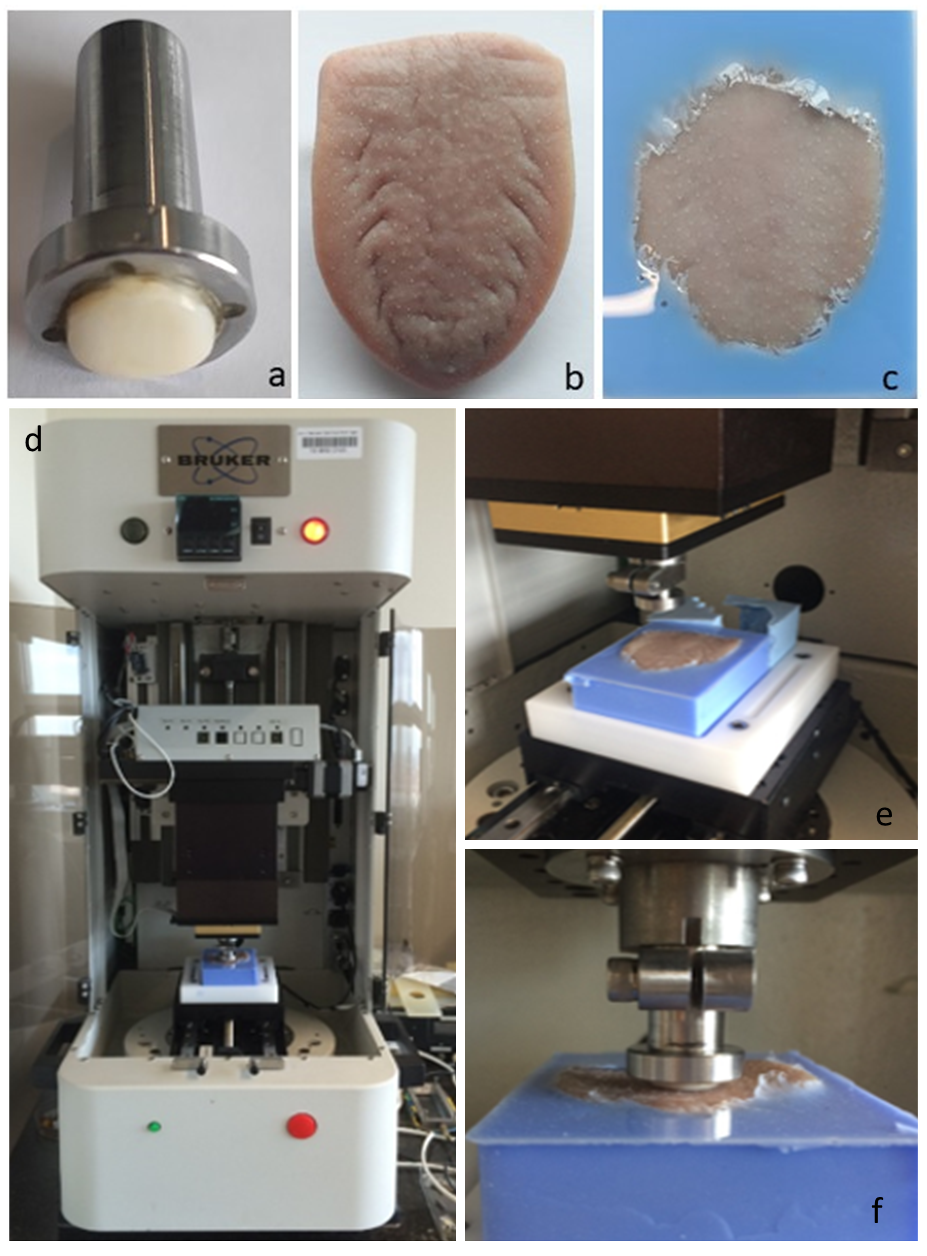


**Supplementary Fig. 6 Tongue-enamel friction system.** [3] **(a) Spherically polished bovine teeth in a metal holder. (b) Fresh porcine tongue. (c) The porcine tongue moulded in duplicating silicone. (d) The tongue and enamel fixed in the universal mechanical tester. (e) Magnification of the lubrication part. (f) Close up of the tongue-enamel in contact. The sliding direction is back and forth in respect to (f). The sliding distance was set on 10 mm and the sliding velocity at 4 mm/s. The normal load was set on 0.25 N. The various volumes of whole saliva were applied directly on the tongue-enamel interface. Using the same enamel and sliding distance ensured a same sliding interface area of 233 mm^2^ for all experiments. The enamel was cleaned after each experiment by rubbing the surface against a wetted polishing cloth** **with 0.05-micron alumina micro-polish (Buehler, Lake Bluff, IL, USA) to remove attached proteins. Hereafter the enamel was rinsed with demineralized water and sonicated for 5 min**

***Supplementary Table 3.*** *The table summarizes the xerogenic medication used and the number of patients in the before radiotherapy, after radiotherapy and primary Sjögren’s syndrome groups that used the medication.*

| **Medication with xerogenic effects** | **Before RT** | **After RT** | **pSS** | **Medication with xerogenic effects** | **Before RT** | **After RT** | **pSS** |
| --- | --- | --- | --- | --- | --- | --- | --- |
| **Alfuzosin** |  |  | 1 | **Methylphenidate** |  |  | 1 |
| **Amitriptyline** |  |  | 1 | **Metronidazole** |  |  | 1 |
| **Amlodipine** | 2 | 1 | 1 | **Metoprolol** | 4 | 3 | 2 |
| **Clonidine** | 1 |  |  | **Montelukast** |  |  | 1 |
| **Diazepam** | 3 | 2 |  | **Morphine** |  | 2 |  |
| **Enalapril** | 2 | 1 | 3 | **Naproxen** | 2 | 1 | 7 |
| **Esomeprazole** | 1 |  | 2 | **Omeprazole** | 2 | 4 | 6 |
| **Ethinylestradiol/drospirenone** |  |  | 1 | **Oxazepam** |  |  | 1 |
| **Etoricoxib** |  |  | 3 | **Oxycodone** | 1 | 2 |  |
| **Fentanyl** |  | 1 |  | **Pantoprazole** | 3 | 3 |  |
| **Flecainide** |  |  | 1 | **Promethazine** |  |  | 1 |
| **Fluticasone** |  |  | 1 | **Propranolol** | 2 | 1 |  |
| **Formoterol/beclomethasone** |  |  | 3 | **Rivaroxaban** |  |  | 1 |
| **Furosemide** |  |  | 1 | **Rosuvastatin** |  |  | 1 |
| **Hydrochlorothiazide** | 1 | 1 | 2 | **Scopolamine** |  | 1 |  |
| **Ibuprofen** |  |  | 2 | **Temazepam** |  | 2 |  |
| **Irbesartan** |  |  | 1 | **Varenicline** | 2 | 2 |  |
| **Ketanserine** |  |  | 1 | **Venlafaxine** |  |  | 1 |
| **Levocetirizine** |  |  | 2 |  |  |  |  |

## References

1. Thomson WM, Williams SM (2000) Further testing of the xerostomia inventory. Oral Surg Oral Med Oral Pathol Oral Radiol Endod 89:46–50. https://doi.org/10.1016/S1079-2104(00)80013-X

2. Van der Putten GJ, Brand HS, Schols JMGA, De Baat C (2011) The diagnostic suitability of a xerostomia questionnaire and the association between xerostomia, hyposalivation and medication use in a group of nursing home residents. Clin Oral Investig 15:185–192. https://doi.org/10.1007/s00784-010-0382-1

3. Vinke J, Kaper HJ, Vissink A, Sharma PK (2018) An ex vivo salivary lubrication system to mimic xerostomic conditions and to predict the lubricating properties of xerostomia relieving agents. Sci Rep 8:9087. https://doi.org/10.1038/s41598-018-27380-7
